# In This Issue

**DOI:** 10.1002/cfg.472

**Published:** 2005-04


**Comparative and Functional Genomics**
*Comp Funct Genom* 2005; **6**: 113.
Published online in Wiley InterScience (www.interscience.wiley.com). **DOI:** 10.1002/cfg.472

### In this issue

### Special section of reviews and papers from the Plant and Animal Genomes XIII (PAGXIII) conference

**John Stevens and Rebecca Doerge** describe a statistically-based meta-analytic approach that systematically combines microarray results from the different laboratories to provide a single estimate of the degree of differential expression for each gene.

**Guilherme Rosa and colleagues** review issues relating to the design and analysis of two-colour microarray experiments using mixed effects models, paying special attention to the different hierarchical levels of replication and the consequent effects of these on the use of appropriate error terms for comparing experimental groups.

**Jonas Almeida *et al.*** discuss the potential role for microarrays in environmental biosensing. They describe the requirements and key considerations for success, and the obstacles that remain to be overcome, in particular in the analysis and interpretation of the data obtained.

The Korea *Brassica* Genome Project is sequencing chromosome 1 of *Brassica rapa*. **Tae-Jin Yang and colleagues** describe the approach they are taking for sequencing, and for construction of an integrated physical map to support the Multinational *Brassica* Genome Project (MBGP).

**Paul Beckett *et al.*** describe their computational approach to *Brassica-Arabidopsis* comparative genomics and the datasets that they make available. They also discuss how this field will evolve with the *Brassica* genome sequence data that will come from the MBGP.

**Lukas Mueller and colleagues** introduce the tomato sequencing project, which is the first cornerstone of the International Solanaceae Genome Project (SOL). They describe how the gene-rich regions of the *Solanum lycopersicum* genome will be sequenced by the international consortium.

**Jim Leebens-Mack *et al.*** review the presentations on plant reproductive genomics at PAGXIII, focusing mainly on the Plant Reproductive Genomics Workshop. They highlight recent advances in our understanding of floral development and discuss how new genomic resources for an expanding set of plant species are increasing our knowledge in this area.

**Russell Kohel and John Yu** report on the International Cotton Genome Initiative (ICGI) Workshop. This workshop looked at the experiences of other crop genome programs in establishing their goals and setting up international exchanges, and at the role played so far by ICGI in international collaborations.

**David Henderson and colleagues** report on the joint Cattle and Sheep Workshop. This session profiled current and future technologies for ruminant research, identifying key areas for future efforts, such as bioinformatics, and areas requiring immediate attention (the implementation of research findings and education of producers).

### Conference Calendar

A listing of genomics related conferences planned for July to September 2005.

Copyright © 2005 John Wiley & Sons, Ltd.

